# Debates in Management and Treatment of CMV in Pediatric Solid Organ Transplant Recipients: A Case‐Based Discussion

**DOI:** 10.1111/petr.70135

**Published:** 2025-07-16

**Authors:** Kevin J. Downes, Lara Danziger‐Isakov, Gustavo Varela‐Fascinetto, Betsy C. Herold, Michael Green, Arnaud G. L'Huillier

**Affiliations:** ^1^ Department of Pediatrics Perelman School at Medicine of the University of Pennsylvania Philadelphia Pennsylvania USA; ^2^ Division of Infectious Diseases Children's Hospital of Philadelphia Philadelphia Pennsylvania USA; ^3^ Cincinnati Children's Hospital Medical Center and University of Cincinnati Cincinnati Ohio USA; ^4^ Transplantation Department Hospital Infantil de México Federico Gómez Mexico City Mexico; ^5^ Division of Infectious Diseases, Department of Pediatrics Children's Hospital at Montefiore, Albert Einstein College of Medicine Bronx New York USA; ^6^ Departments of Pediatrics and Surgery UPMC Children's Hospital of Pittsburgh Pittsburgh Pennsylvania USA; ^7^ Departments of Pediatrics and Surgery University of Pittsburgh School of Medicine Pittsburgh Pennsylvania USA; ^8^ Pediatric Infectious Diseases Unit, Department of Woman, Child and Adolescent Health Geneva University Hospitals Geneva Switzerland; ^9^ Department of Pediatrics Gynecology and Obstetrics Faculty of Medicine Geneva Switzerland

## Abstract

The recently published 4th International Consensus Guidelines on the Management of CMV in Solid‐Organ Transplantation offers updated recommendations for CMV prevention, treatment, and monitoring in pediatric solid organ transplant (pSOT) recipients. Although these guidelines are supported by a thorough review of published data, there remain unanswered questions due to a paucity of pediatric data, conflicting findings across studies, or areas where pediatric and adult data are divergent. The current report highlights controversial areas of management, emphasizes the rationale for certain expert opinions, and calls attention to topics that require further research using a case‐based approach.

**Abbreviations**
AUC: area‐under‐the‐curve
BSA: body surface area
BW: body weight
CMV: cytomegalovirus
CrCl: creatinine clearance
D: donor
GCV: ganciclovir
PK: pharmacokinetic
pSOT: pediatric solid organ transplant
qNAT: quantitative nucleic acid testing
R: recipient
valGCV: valganciclovir

## 1 Introduction

Despite important advances in prevention, treatment, and monitoring, cytomegalovirus (CMV) remains an important cause of morbidity in pediatric solid organ transplant (pSOT) populations. Antiviral prophylaxis and preemptive treatment strategies have limited the burden of CMV disease in pSOT, and standardization of quantitative nucleic acid testing (qNAT) across laboratories has promoted implementation of more effective monitoring strategies. Additionally, the development of newer antiviral agents and adjunctive treatments, such as virus‐specific T cells, holds promise for safer and more durable prophylactic and therapeutic options. Yet, important knowledge gaps regarding CMV management persist and pose challenges to clinicians and researchers in the field.

The recently published “4th International Consensus Guidelines on the Management of CMV in Solid‐Organ Transplantation” (hereby referred as the CMV‐TTS Consensus Guidelines) offers updated recommendations for CMV prevention, treatment, and monitoring in pSOT, supported by a thorough review of published data [1]. While this document includes pediatric‐specific guidance, supportive data from multicenter pediatric studies remain limited in a variety of areas. Further, extrapolation from adult experiences may be inappropriate when managing pediatric transplant recipients, who are by no means little transplanted adults.

The goal of this manuscript is to present some of the key questions that remained unanswered during the development of these guidelines either because there is a paucity of available pediatric data, conflicting findings across studies, or areas where pediatric and adult data are divergent. As members of the pediatric group within The Transplantation Society International CMV Consensus Group, we aimed to highlight controversial areas of management, emphasize the rationale for certain expert opinions, and call attention to topics that require further research using a case‐based approach.

## 2 Case 1: What Is the Rationale for CMV DNAemia Monitoring During Prophylaxis and How Should One Respond to Breakthrough DNAemia?

*A CMV‐seronegative 4‐year‐old with a history of biliary atresia received a liver from a CMV‐seropositive donor (D+/R−). Antiviral prophylaxis with valganciclovir (valGCV) is prescribed for an anticipated duration of 3 months, but 45 days post‐transplant, the recipient has a positive quantitative plasma CMV PCR test at 1500 IU/mL, which was obtained as part of routine surveillance. There are no associated symptoms or signs of CMV disease*.

### 2.1 Why Was Monitoring Performed During Prophylaxis?

While the adult literature does not support monitoring during prophylaxis, several cohort studies report breakthrough CMV DNAemia during valGCV prophylaxis in pSOT recipients and suggest that breakthrough DNAemia may be associated with adverse outcomes [2]. Breakthrough CMV DNAemia has been reported in pSOT recipients ranging from 1.5% to 22.5% depending on the organ (Table 1). Breakthrough CMV DNAemia occurred more frequently in pediatric kidney recipients with CMV seropositive donors (D+) [6] and heart recipients with CMV sero‐mismatch (donor seropositive and recipient seronegative D+/R‐) [8]. The impact of monitoring is highlighted by Pangonis et al., who found that breakthrough increased from 1.6% to 8.5% during an intervention that increased screening frequency [5]. As breakthrough DNAemia does not commonly progress to CMV disease, one might argue not to monitor during prophylaxis. However, as described further below, breakthrough DNAemia may be associated with other adverse outcomes, including organ rejection, other infections, and the development of valGCV/ganciclovir (GCV) resistance, although the data are controversial, and direct causality has not been demonstrated. The optimal frequency of monitoring while receiving antiviral prophylaxis is unknown, but typically ranges from every 2–4 weeks. In addition, if a patient develops symptoms compatible with CMV syndrome/disease, viral load should be measured.

**TABLE 1:** Incidence of breakthrough CMV DNAemia across organs.

		Organ(s)	Rate(s)	Comments
Liverman et al. [3]	Single center (*n* = 393)	Kidney Liver Heart	6.7% 10.3% 15.6%	8% if exclude those with antiviral dose‐adjustment
Downes et al. [4]	Single center (*n* = 271)	Kidney/Liver/Heart/Lung	4.8%	
Pangonis et al. [5]	Single center (*n* = 380)	Kidney/Liver/Heart	5%	16/19 events occurred within increased surveillance cohort
Paulsen et al. [6]	Single center (*n* = 92)	Kidney	15%	Routine monitoring performed
Levi et al. [7]	Single center (*n* = 68)	Kidney	1.5%	
Das et al. [8]	Single center (*n* = 97)	Heart	14% D+/R 10% R+	
Knackstedt et al. [9]	Multi‐center (*n* = 199)	Liver	6.5% D+/R‐ 8.4% R+	Prospective cohort
Foca et al. [2]	Multi‐center (*n* = 749)	Kidney Liver Heart Lung	4.8% 17% 11.2% 22.5%	Monitoring varied by institution and organ
Valencia Deray et al. [10]	Single center (*n* = 687)	Kidney Liver Heart Lung Multi‐organ	0% 13.5% 3.3% 13.8% 14.3%	

### 2.2 When Should I Intervene?

Monitoring for clinically silent breakthrough DNAemia can only be justified if results of the lab tests affect management, including increasing prophylaxis to treatment dosing or modifying immunosuppression. The question about when and how to intervene is difficult as there have been no randomized studies, and, as noted above, CMV DNAemia may be transient and resolve without progression to CMV syndrome or end‐organ disease. In fact, CMV syndrome and end‐organ disease were infrequently reported during prophylaxis [2, 3, 5, 11]. For example, breakthrough CMV disease occurred in 2.3% of a large cohort of pSOT recipients [2]. Even though the absolute frequency of breakthrough CMV disease is rare, the proportion of breakthrough cases presenting as CMV syndrome and end‐organ disease ranges between 4% and 29% [4, 6, 10] and 0%–13% [4, 6, 10], respectively. Furthermore, data from several studies evaluating pre‐emptive therapy in pediatric liver transplant recipients where CMV monitoring determines initiation of antiviral therapy suggest that the presence of CMV DNAemia at low levels may not require specific intervention. The presence of CMV DNAemia or pp65 antigenemia in these studies ranged from 61% to 71% during monitoring [12, 13, 14]; however, antiviral therapy was only administered in 31%–63% of positive CMV events without evidence of progression in those not treated. Reasons for treatment initiation included high‐risk status (CMV D+/R−), higher viral load (some studies have used a threshold of above 1500 copies/mL in whole blood [14] or ≥ 2000 IU/mL in plasma [12] even though there is no accepted cut‐off), intense net state of immunosuppression, time since SOT, lymphopenia, and the presence of CMV syndrome or disease at the time CMV DNA is detected. Together, these data suggest that breakthrough CMV below these low levels should be monitored for persistence or progression with serial CMV DNA testing rather than reflexively increasing antiviral therapy to treatment dosing. However, careful monitoring of breakthrough DNAemia is warranted as it may reflect the emergence of valGCV/GCV resistance, which is documented in 10%–25% of breakthrough DNAemia cases [3, 15]. As letermovir prophylaxis expands beyond adult kidney transplant recipients, the issue of CMV monitoring may become more complex as CMV DNA “blips” may occur more frequently. These noninfectious transient episodes of CMV DNAemia may reflect the fact that letermovir blocks a late stage in viral replication (cleavage and packaging of DNA) rather than the DNA polymerase, which prohibits early viral replication [16].

### 2.3 Are There Indirect Effects of CMV DNAemia That Support Treatment?

In addition to preventing progression to CMV syndrome or end‐organ disease, some advocate treating breakthrough CMV DNAemia because of the potential indirect effects of ongoing viral replication. These include increased rates of graft rejection and an association with other infections, possibly a consequence of the immunomodulatory effects of CMV. However, the pediatric data are controversial. For example, in a multicenter study conducted by the Pediatric Infectious Disease Transplant Network, rejection occurred more often in those who experienced breakthrough CMV DNAemia compared to those who did not (35.3% vs. 20.6%, *p* = 0.002) and this association was strongest for the liver transplant cohort [2]. However, whether CMV DNAemia preceded rejection or vice versa could not be determined. Similarly, in a large single‐center retrospective study of pSOT recipients receiving universal prophylaxis, CMV DNAemia (which occurred most often after completion of primary prophylaxis) was also associated with rejection but only in liver transplant recipients [10]. Rabbani et al. found that CMV seropositivity in either the donor or recipient was associated with decreased graft survival using a national database of pediatric heart transplant patients, but the study did not specifically look at episodes of CMV DNAemia [17]. However, other studies have not documented any association between CMV and graft rejection (Table 2).

**TABLE 2:** Clinical studies on potential indirect effects of CMV in pediatric SOT recipients by organ transplanted.

Organ	Author/year	Type, years, size of study	Findings
Kidney	Smith et al. [18]	Single center Prospective 2000–2005 *n* = 55	CMV DNAemia associated with decreased renal function at 2 years post‐transplant and development of moderate to severe interstitial fibrosis and tubular atrophy
	Yamada et al. [19]	Single center Retrospective 1973–2010 *n* = 104	Chronic allograft insufficiency, which was identified as major risk factor for graft loss, correlated with CMV infection (pp. 65 antigenemia)
	Hocker et al. [20]	Multicenter registry Retrospective 2000–2013 *n* = 242	CMV antiviral prophylaxis associated with preservation of transplant function 3 years post‐transplant. CMV replication (DNAemia or pp65 antigenemia) associated with decline in graft function
	Ettenger et al. [21]	Multicenter Prospective 2009–2013 *n* = 106	Pretransplant CMV serostatus and DNAemia were NOT related to biopsy‐proven acute rejection or de novo donor‐specific antibody formation
	Gotoh et al. [22]	Multicenter (Japan) Retrospective 2011–2014 *n* = 163	CMV infection or disease (pp65 antigenemia) not associated with acute rejection, renal function or other infections at 2 years post‐transplant
	Oomen et al. [23]	Single center Retrospective 2002–2018 *n* = 100	No association between CMV (DNAemia) and graft function in the first 5 years post‐transplant
Heart	Hussain et al. [24]	Single center Retrospective 1989–2003 *n* = 165	Pre‐transplant CMV seropositivity associated with coronary artery disease, all‐cause mortality and coronary death
	Mahle et al. [25]	Multicenter Retrospective 1993–2007 *n* = 1598	No association between pre‐transplant CMV seropositivity and coronary artery vasculopathy or mortality
	Das et al. [8]	Single center Retrospective 2010–2016 *n* = 91	No association between CMV risk stratification and overall graft loss but CMV high risk status associated with decreased rejection‐free survival compared to intermediate or low risk groups
	Rabbani et al. [17]	Multicenter registry Retrospective 1987–2015 *n* = 4968	CMV seropositive transplants who received no antiviral prophylaxis had impaired graft survival compared to those who received antivirals or seronegative recipients
Liver	Indolfi et al. [26]	Single center Retrospective 2007–2008 *n* = 62	No association between seropositivity before transplantation or CMV DNAemia after transplantation with rejection
	Furuichi et al. [27]	Single center Retrospective 2005–2015 *n* = 337	CMV pp65 antigenemia was associated with rejection, but ~ 60% of rejection episodes were prior to CMV
Liver
	Nicastro et al. [13]	Single center Retrospective 2008–2014 *n* = 100	No association between CMV and Epstein‐Barr virus infection (DNAemia), sepsis, biliary and vascular complications
	Arroyo‐Orvananos et al. [14]	Single center Retrospective 1998–2018 *n* = 118	No association between CMV infection or disease (pp65 antigenemia or DNAemia) with acute or chronic rejection, survival, fungal infection, EBV infection, PTLD or biliary complications
Chanburanavah et al. [12]	Single center Retrospective 2001–2020 *n* = 126	Association between CMV infection (DNAemia) and acute cellular rejection. No effect of CMV infection on EBV infection or development of PTLD
Lung	Danziger‐Isakov et al. [28]	Multicenter Retrospective 1988–2005 *n* = 555	CMV D+/R‐ serostatus was associated with pulmonary fungal infections
	Danziger‐Isakov et al. [29]	Multicenter Retrospective 2004–2007 *n* = 577	CMVIG administration was associated with increased mortality in the first year post‐transplant but was not associated with acute rejection, respiratory fungal or viral infections
	Liu et al. [30]	Multicenter Retrospective 1988–2005 *n* = 576	CMV serostatus or infection was not associated with respiratory viral infections
All organs	Valencia Deray et al. [10]	Single center Retrospective 2011–2018 *n* = 687	CMV DNAemia was associated with rejection in liver transplant recipients only but not with mortality or other organ‐specific adverse outcomes
	Foca et al. [2]	Multicenter Retrospective 2016–2019 *n* = 749	Breakthrough CMV DNAemia was associated with rejection with highest risk among liver transplant recipients. Bacteremia, adenovirus and EBV DNAemia were more common in patients with breakthrough CMV DNAemia compared to no CMV

*Note:* Adapted from Kotton et al. [1].

There has also been a suggestion that CMV reactivation is associated with increased risk for other infections. Initially, this was described in adults where *Pneumocystis jirovecii* pneumonia was more severe in patients who also experienced CMV reactivation [31]. Pediatric studies have not shown a similar association, but it has been suggested that CMV is associated with increased rates of other infections, including bacteremia, EBV, and adenovirus DNAemia [2]. Again, causality cannot be inferred from this study as the timing of infections, impact of valGCV‐associated leukopenia, and other potential confounders could not be disentangled. Thus, this is an area that warrants further investigation.

Based on the increased rates of breakthrough DNAemia in children compared to adults and the potential, albeit infrequent, risk for progression to CMV disease, particularly when DNAemia persists and the association between CMV DNAemia and rejection, other infections, and potential development of valGCV/GCV resistance, monitoring during prophylaxis for pediatric SOT recipients is suggested in the current TTS‐CMV Consensus Guidelines [1]. However, there is a clear need for additional research, particularly focused on determining whether there is any causality underlying these observed associations.

Knowledge gaps:

- Indication for monitoring while receiving antiviral prophylaxis (all organs vs. specific situations [e.g., D+/R− only]).
- Optimal frequency and duration of monitoring while receiving antiviral prophylaxis.
- Impact of low‐level DNAemia on duration of prophylaxis and decision to initiate treatment dosing.
- Viral load threshold for treatment initiation.

## 3 Case 2: What Dosing Strategy to Use for CMV Prevention?

*A 6‐year‐old female with end stage kidney disease secondary to nephrotic syndrome is awaiting a live donor transplant from her mother. During pre‐transplant screening, it is determined that the mother is CMV IgG+, while the child is CMV IgG−. The transplant team is planning to start the child on valGCV following transplant for CMV prevention, and the resident asks how dosing of the drug will be determined*.

Currently, there are two approaches to dosing of valGCV in pediatric SOT: one based on body surface area (BSA) and creatinine clearance (CrCL) and the other based on body weight (BW). BSA dosing is recommended on the Food and Drug Administration (FDA) label with the dose calculated according to the equation: 7 × BSA × CrCL. The Mosteller equation is used to calculate BSA [32] while the modified Schwartz equation is used to determine CrCL [33]; a maximum CrCL of 150 mL/min/1.73 m^2^ is recommended when performing CrCL calculations, although we will return to this recommendation later. Early data in support of BSA dosing came from pharmacokinetic (PK) studies conducted in pSOT recipients based on matching of GCV area‐under‐the‐curve (AUC) to AUCs achieved with IV GCV in children and adults [34, 35]. Weight‐based dosing (15–18 mg/kg), on the other hand, was first derived by extrapolating the adult dose of 900 mg/day to children, based on the authors' assumption that the average adult transplant recipient weighs 50–60 kg [36].

**FIGURE 1:**
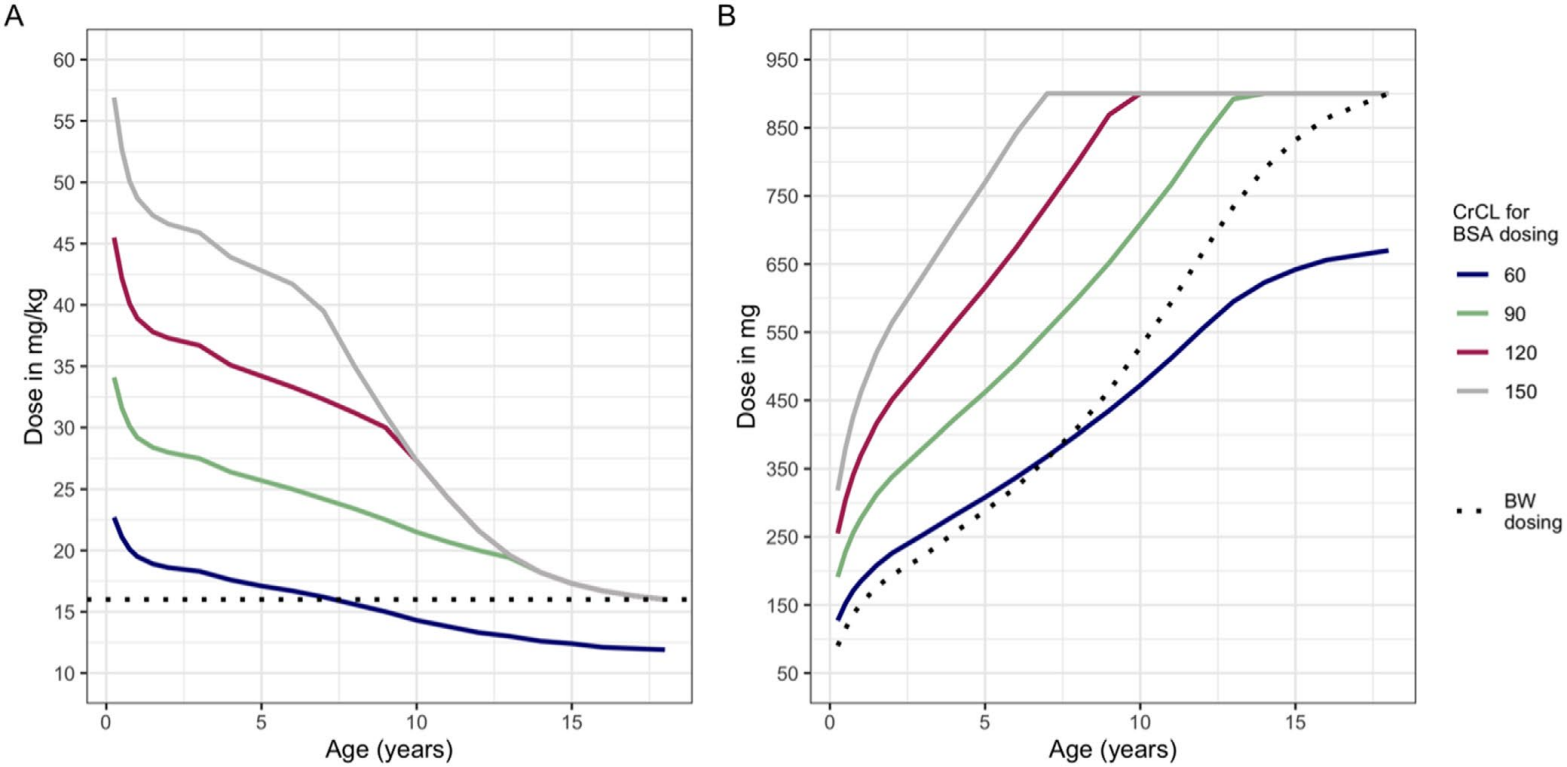
Comparisons of body surface area and body weight dosing of valganciclovir in children. Calculations of body surface area determined using U.S. CDC growth curves and 50th percentile weights and heights/lengths for girls. Solid lines reflect different CrCL (blue = 60 mL/min/1.73 m^2^; green = 90, red = 120, gray = 150 [maximum recommended for calculations according to FDA label]). Dotted line represents body weight‐based dosing, with 16 mg/kg used for all body weight‐based calculations. (A) Dosing in mg/kg and (B) Dosing in mg/dose.

BSA and BW dosing calculations can result in different doses in most children (Figure 1), with BSA‐based doses exceeding those in BW dosing for young children with normal renal function. As might be expected, studies have found that BSA dosing results in higher GCV exposures (i.e., AUC) and more frequent attainment of the putative adult AUC_0‐24_ target of 40–60 mg*h/L for prophylaxis [37, 38, 39, 40]. However, the pharmacokinetics of valGCV/GCV are highly variable in children, and even use of BSA‐based dosing may lead to AUCs above or below these ranges in many children. Furthermore, BSA dosing's greater exposures may contribute to its more frequent toxicities, most notably leukopenia and neutropenia, potentially leading to more frequent discontinuation of therapy, as observed in recent studies including a multicenter study [11, 41]. Although no prospective comparative studies have been performed, rates of breakthrough CMV DNAemia reported during prophylaxis have been comparable across the two dosing strategies [11, 41].

Part of the debate surrounding valGCV dosing has to do with the therapeutic target itself. The AUC_0‐24_ target range of 40–60 mg*h/L was first established in adults with HIV and subsequently confirmed as the goal to achieve viral suppression in adult transplant recipients [42], but this target has never been validated in children. It is theoretically possible that lower AUCs would effectively prevent CMV infection in pSOT recipients, although inappropriately low exposures may contribute to the development of valGCV/GCV resistance. Similarly, a GCV AUC above 60 mg*h/L does not guarantee toxicity. Myelosuppression may be in part dose‐dependent, but the duration of exposure and other intrinsic (e.g., rates of cellular kinase drug phosphorylation and phosphatase dephosphorylation to allow for drug clearance) or extrinsic (e.g., exposure to other myelosuppressive drugs) host factors likely contribute to the individual risk of toxicity. While it is appealing to think of using personalized valGCV dosing in children with therapeutic drug monitoring, it is challenging to do so without a firm therapeutic target.

As previously stated, the US‐FDA approved dosing strategy of valGCV for children is based on BSA and CrCL. The original algorithm for BSA‐dosing of valGCV published in 2009 [34, 35], was developed based on the estimation of CrCl using the modified Schwartz formula [33] and the Jaffe method of measuring serum creatinine (different *K* according to age group). In 2010, the FDA released a safety alert to prevent potential valGCV overdosing in small children with low BW, low BSA and below normal serum creatinine, and prompted modifying the maximal CrCl calculation to 150 mL/min. The Jaffe method is a colorimetric assay that is no longer in use because it gives higher values of serum creatinine, specifically when serum creatinine is low. Most centers now use enzymatic assays that yield lower values of serum creatinine and if used with the original Schwartz formula, overestimate the CrCl by 20%–25%. This may have a profound effect on the calculated dose of valGCV. Schwartz himself modified his formula in 2009 to adjust for the current enzymatic creatinine method. The “updated bedside Schwartz formula” uses a *K* of 0.413 for all ages [43, 44, 45]. Current US national guidelines recommend that the estimated GFR be reported for children using the updated Schwartz “bedside” formula [45]. The most recent version of the valgCV prescribing package insert states that the “*K* values used to calculate CrCL may require correction when enzymatic methods are used”. The multicenter, open‐label study that evaluated the tolerability of 200 days of valGCV prophylaxis in pediatric kidney transplant recipients also recommends the use of the updated bedside equation that uses a *K* of 0.413 for all ages [46].

Table 3 shows how, just by using a different *k* for CrCl calculation in our patient, the dose of oral valGCV is reduced by 25% over a wide range of weight, height, and creatinine values. This would be even more important for adolescent boys 13–20 years old, where the *k* should be reduced from 0.7 to 0.413. By using this more appropriate dose, the myeloid toxicity of valGCV may be reduced while preserving its therapeutic effect. Therefore, when using the BSA method for calculating valGCV dose, it is crucial to not only cap CrCl to 150 mL/min/1.73 m^2^, but to also use the appropriate *k* of 0.413 for all ages.

**TABLE 3:** BSA‐dosing for VALGCV in a 6‐year‐old female using different *k* values.

CDC percentile	Weight (kg)	Height (cm)	BSA	Creatinine (mg/dL)	CrCl (mL/min/1.73 m^2^)		Dose valGCV (mg)		Dose overestimation (%)
					*k* = 0.55	*k* = 0.413	*k* = 0.55	*k* = 0.413	
5th	15.7	105	0.68	0.3	150.00^a^	144.55	711	685	3.8^a^
				0.5	115.50	86.73	547	411	33.2
				0.7	82.50	61.95	391	293	33.2
				1	57.75	43.37	274	205	33.2
50th	20.2	114.8	0.80	0.3	150.00^a^	150.00^a^	843	843	0.0^a^
				0.5	126.28	94.82	709	533	33.2
				0.7	90.20	67.73	507	381	33.2
				1	63.14	47.41	355	266	33.2
95th	29.6	125.7	1.02	0.3	150.00^a^	150.00^a^	1067	1067	0.0
				0.5	138.27	103.83	984	739	33.2
				0.7	98.76	74.16	703	528	33.2
				1	69.14	51.91	492	369	33.2

^a^ CrCl cap to 150 mL/min/1.73 m^2^.

Given the lack of any controlled randomized studies comparing either the efficacy or safety of BSA vs. BW dosing, the consensus guidelines did not specifically recommend one approach versus another. While the recent multicenter study favored BW dosing with respect to the risk for myelosuppression, there were no significant differences in rates of breakthrough DNAemia. Moreover, it was not randomized and retrospective, thus limiting definitive conclusions. Similarly, in the absence of pediatric data on target AUC to prevent CMV, or drug concentrations required to prevent emergence of resistance, therapeutic drug monitoring is currently not recommended. However, it is noteworthy that the pharmacokinetics of valGCV demonstrate substantial inter‐individual variability in children, meaning that the drug concentrations (and AUC) vary widely across individuals regardless of the dosing strategy used. Therefore, clinicians should be attuned to the possibility that breakthrough infection while receiving antiviral prophylaxis may be due to inadequate dosing in that patient. Without the use of therapeutic drug monitoring, dose adjustment to optimize GCV AUC is not possible. Ultimately, it is unlikely that a randomized trial will ever be conducted, but a larger prospective study collecting data from transplant programs that choose to use BW vs. BSA dosing would be insightful.

Knowledge gaps:

- Establishment of a ganciclovir AUC0‐24 target range in pSOT
- Benefits of therapeutic drug monitoring for (val)ganciclovir in pediatric SOT

## 4 Case 3: Treatment of CMV Refractory Infection

*One month after completion of 100 days of CMV prophylaxis, a 13‐year‐old liver transplant recipient develops CMV DNAemia with a CMV plasma viral load of 35 000 IU/mL. Despite now receiving adequate treatment dosing and compliance with oral valGCV for 15 days, there is no reduction in viral load. What would be your next steps in treatment management?*

This patient was appropriately started on oral valGCV for treatment of CMV infection as recommended in the TTS‐CMV Consensus Guidelines [1]. Decline in CMV DNA viral load and clinical symptoms may be delayed after the initiation of treatment with oral valGCV and/or IV GCV [47]. However, patients with persistent symptoms and CMV viral loads that have not begun to improve despite ≥ 14 days of appropriate therapy are considered to have a refractory infection [48]. The initial response in this setting is to reduce immunosuppression if feasible and to consider IV GCV if the patient is still receiving oral therapy. In addition, cumulative valGCV/GCV administration of 4 weeks or more (including prior prophylaxis) increases the risk for valGCV/GCV resistance. Therefore, alternative drugs should be considered and a blood specimen for genotypic resistance testing should be sent to attempt to identify the presence of resistance genotypes [1], which can definitively inform subsequent treatment decisions.

With regard to treatment, for this 13‐year‐old patient, the updated CMV‐TTS Consensus Guidelines recommend replacing valGCV/GCV with maribavir as first‐line therapy for refractory or valGCV/GCV‐resistant CMV for adults and children who are at least 12 years of age and weigh at least 35 kg, even though there are no clinical data in children to recommend maribavir over foscarnet in the case of refractory or valGCV/GCV‐resistant CMV. However, as viral loads > 50 000 IU/L increase the likelihood of maribavir resistance, foscarnet may be preferred in those instances [49]. The switch of antivirals can be done empirically while awaiting resistance testing results. This recommendation is based on high‐level evidence of clinical success with a favorable safety profile and lower risk of cross‐resistance in studies of refractory/resistant CMV in adult SOT recipients [50]. Extension of both the approval of maribavir and the recommendation for its use in children 12 years of age and older is based on PK modeling data demonstrating that adult dosing could be extended to adolescents [51] as no participants enrolled in the SOLSTICE trial were under 18 years of age. Pediatric‐specific data for treatment of resistant/refractory CMV infection are not currently available although a non‐randomized open‐label study in pediatric immunocompromised patients aiming to address maribavir age‐specific pharmacokinetics and safety questions is underway (NCT 05319353). Second‐line alternatives for resistant/refractory infection include foscarnet or cidofovir. Both these second‐line agents, however, are nephrotoxic, whereas there is no evidence of nephrotoxicity or bone marrow suppression with maribavir. One limitation to maribavir is that it is metabolized by CYP3A4 and thus immunosuppressive drug levels should be monitored because of concerns for drug–drug interactions. Further, maribavir demonstrates suboptimal penetration into the CNS and eye and, thus, has a limited role in the treatment of infections involving these sites.

Adjunctive intravenous immunoglobulins or CMV‐specific immunoglobulins are not recommended based on a lack of supportive data, although some may consider prescribing them in the setting of refractory disease. However, they could be considered in certain circumstances, for instance, in patients with concurrent hypogammaglobulinemia. Similarly, whenever available, the use of cellular therapies (such as CMV‐specific or multivirus‐specific T‐cell therapy) can be considered in this context, particularly if alternative antiviral treatments cannot be tolerated (i.e., foscarnet or cidofovir in a child with acute/chronic kidney failure), were ineffective, or are not accessible [1]. These therapies should be pursued under formal research protocols whenever feasible. In the absence of validated markers of the net state of immunosuppression, identifying patients who might benefit the most from these adjunctive treatment strategies remains a clinical challenge.

### 4.1 What Would You Do Differently if the Child Was 8 yo?

In children < 12 years of age or weighing < 35 kg, there is a lack of PK, safety, and efficacy data regarding maribavir administration, and therefore dosing is not established. As a consequence, its use is not routinely recommended in this setting, making foscarnet first‐line therapy in case of refractory or resistant infection [1]. Cidofovir could be considered an alternative but is associated with significant rates of toxicity.

After several weeks of maribavir treatment, there is a complete clinical and virological response, allowing for treatment discontinuation. Phenotypic testing has not shown any mutation known to confer resistance to antiviral agents. Would you consider secondary prophylaxis? If yes, which factors should be taken into decision‐making?

Use of secondary prophylaxis is suggested for children with repeated episodes of either CMV DNAemia requiring treatment or CMV disease [1]. Use of secondary prophylaxis should also be considered after the first episode of CMV disease [1], which is the situation applicable to the current scenario. In this situation, several factors should be considered in the decision‐making. The first and foremost factor to be considered is the net state of immunosuppression, as it impacts the likelihood of relapse and consequently, progression to organ disease. Some adult experts advocate testing for CMV specific T cell responses to help guide this decision process, although pediatric data in support of this practice are absent. Other factors to be considered include: (1) how primary prophylaxis was tolerated in terms of drug toxicity, (2) whether the patient is currently or expected to be in the near future under intensified immunosuppression for rejection, (3) whether any mutation conferring valGCV/GCV resistance was identified, and (4) whether the patient will be able to comply with routine monitoring after treatment. Once a decision to use secondary prophylaxis has been made, there are no data to suggest a specific duration [1]; however, decision‐making should take into account the immunosuppression regimen, patient age, presence of other opportunistic infections, and other risk factors.

Knowledge gaps:

- Comparison of maribavir versus foscarnet as a first‐line agent in case of resistant or refractory infection.
- Role of immunoglobulins and cellular therapies in resistant and refractory infection.
- Indications and duration of secondary prophylaxis (establishment of a viral load threshold to initiate secondary prophylaxis; how to address intermittent DNAemia blips).

## 5 Conclusions

The CMV‐TTS Consensus Guidelines provide guidance on CMV management and prevention in pediatric SOT recipients. Although some clinical trials have been performed in adult transplant recipients, offering some comparative data on treatment and monitoring strategies, most pediatric data are single center and descriptive. Thus, there remain areas of the field for which the best, safest, and most effective strategies are still unknown. We comment on a few such areas above, highlighting some of the nuances not fully elaborated upon within the guidelines. We hope that this provides additional support for the recommendations offered within the guidelines on a few of the more highly debated questions. Moreover, we implore the pediatric transplant community to continue to address the research gaps through rigorous, prospective (ideally) studies.

## Conflicts of Interest

K.J.D. has received research funding to his institution from Merck Inc., Paratek Inc., and Veloxis Pharmaceuticals Inc. L.D.I. has received consultancy fees from Astellas and Takeda. Her institution received support for contracted clinical research from Ansun BioPharma, AiCuris, Astellas, Merck, Pfizer, and Takeda. G.V.‐F. has received consultancy fees from Novartis, Asofarma, and research grants from Roche. B.C.H. is a consultant for Eurofins‐Viracor (clinical advisory board). M.G. is a consultant for ITB‐MED (DSMB member), Bristol Myers Squibb (DSMB member) and ADMA (advisory board). A.G.L. is a consultant for Sanofi (advisory board) and MSD (advisory board). None of these overlap with current work.

## Data Availability

Data sharing is not applicable to this article as no new data were created or analyzed in this study.

## References

[1] C. N. Kotton , D. Kumar , O. Manuel , et al., “The Fourth International Consensus Guidelines on the Management of Cytomegalovirus in Solid Organ Transplantation,” Transplantation 109 (2025): 1066–1110, 10.1097/TP.0000000000005374. 40200403 PMC12180710

[2] M. Foca , S. Demirhan , F. M. Munoz , et al., “Multicenter Analysis of Valganciclovir Prophylaxis in Pediatric Solid Organ Transplant Recipients,” Open Forum Infectious Diseases 11, no. 7 (2024): ofae353, 10.1093/ofid/ofae353. 38979014 PMC11229698

[3] R. Liverman , A. Serluco , G. Nance , et al., “Incidence of Cytomegalovirus DNAemia in Pediatric Kidney, Liver, and Heart Transplant Recipients: Efficacy and Risk Factors Associated With Failure of Weight‐Based Dosed Valganciclovir Prophylaxis,” Pediatric Transplantation 27, no. 4 (2023): e14493, 10.1111/petr.14493. 36945819

[4] K. J. Downes , A. Sharova , C. L. K. Boge , et al., “CMV Infection and Management Among Pediatric Solid Organ Transplant Recipients,” Pediatric Transplantation 26, no. 3 (2022): e14220, 10.1111/petr.14220. 34994041

[5] S. Pangonis , G. Paulsen , H. Andersen , et al., “Evaluation of a Change in Cytomegalovirus Prevention Strategy Following Pediatric Solid Organ Transplantation,” Transplant Infectious Disease 22, no. 2 (2020): e13232, 10.1111/tid.13232. 31840369

[6] G. Paulsen , P. Cumagun , E. Mixon , K. Fowler , D. Feig , and M. Shimamura , “Cytomegalovirus and Epstein‐Barr Virus Infections Among Pediatric Kidney Transplant Recipients at a Center Using Universal Valganciclovir Prophylaxis,” Pediatric Transplantation 23, no. 3 (2019): e13382, 10.1111/petr.13382. 30786115 PMC6650320

[7] S. Levi , M. Davidovits , H. Alfandari , et al., “EBV, CMV, and BK Viral Infections in Pediatric Kidney Transplantation: Frequency, Risk Factors, Treatment, and Outcomes,” Pediatric Transplantation 26, no. 3 (2022): e14199, 10.1111/petr.14199. 34817112

[8] B. B. Das , B. K. Prusty , J. Niu , and P. K. Sue , “Cytomegalovirus Infection and Allograft Rejection Among Pediatric Heart Transplant Recipients in the Era of Valganciclovir Prophylaxis,” Pediatric Transplantation 24, no. 8 (2020): e13750, 10.1111/petr.13750. 32573886

[9] E. D. Knackstedt , S. G. Anderson , R. Anand , et al., “Cytomegalovirus (CMV) Prophylaxis in Pediatric Liver Transplantation (PLT): A Comparison of Strategies Across the SPLIT Consortium,” American Journal of Transplantation 25, no. 5 (2024): 1098–1106, 10.1016/j.ajt.2024.09.025. 39368657

[10] K. G. Valencia Deray , K. E. Hosek , D. Chilukuri , et al., “Epidemiology and Long‐Term Outcomes of Cytomegalovirus DNAemia and Disease in Pediatric Solid Organ Transplant Recipients,” American Journal of Transplantation 22, no. 1 (2022): 187–198, 10.1111/ajt.16822. 34467658

[11] S. Demirhan , F. M. Munoz , K. G. Valencia Deray , et al., “Body Surface Area Compared to Body Weight Dosing of Valganciclovir Is Associated With Increased Toxicity in Pediatric Solid Organ Transplantation Recipients,” American Journal of Transplantation 23, no. 12 (2023): 1961–1971, 10.1016/j.ajt.2023.07.013. 37499799

[12] N. Chanburanavah , S. Boonsathorn , N. Apiwattanakul , et al., “Risk Factors of Cytomegalovirus Infection After Pediatric Liver Transplantation and Effectiveness of Preemptive Therapy,” Transplant Infectious Disease 25, no. 3 (2023): e14057, 10.1111/tid.14057. 37013827

[13] E. Nicastro , S. Giovannozzi , P. Stroppa , et al., “Effectiveness of Preemptive Therapy for Cytomegalovirus Disease in Pediatric Liver Transplantation,” Transplantation 101, no. 4 (2017): 804–810, 10.1097/TP.0000000000001531. 27755504 PMC7228596

[14] J. Arroyo‐Orvananos , J. A. Hernandez‐Plata , R. Erro‐Aboytia , et al., “Cytomegalovirus Infection and Disease in Pediatric Liver Transplantation: Burden of Disease Under a Preemptive Therapy Approach,” Pediatric Transplantation 27, no. 1 (2023): e14356, 10.1111/petr.14356. 35842927

[15] M. P. Khurana , I. P. Lodding , A. Mocroft , et al., “Risk Factors for Failure of Primary (Val)ganciclovir Prophylaxis Against Cytomegalovirus Infection and Disease in Solid Organ Transplant Recipients,” Open Forum Infectious Diseases 6, no. 6 (2019): ofz215, 10.1093/ofid/ofz215. 31211159 PMC6559280

[16] A. T. Payne , B. K. Lindner , A. J. Gilbert , R. N. Kumar , B. S. Thomas , and J. G. Timpone , “Evaluation of Cytomegalovirus “Blips” in High‐Risk Kidney/Kidney‐Pancreas Transplant Recipients,” Transplant Infectious Disease 24, no. 2 (2022): e13789, 10.1111/tid.13789. 35014122

[17] N. Rabbani , R. A. Kronmal , T. Wagner , et al., “Association Between Cytomegalovirus Serostatus, Antiviral Therapy, and Allograft Survival in Pediatric Heart Transplantation,” Transplant International 35 (2022): 10121, 10.3389/ti.2022.10121. 35368645 PMC8964945

[18] J. M. Smith , L. Corey , R. Bittner , et al., “Subclinical Viremia Increases Risk for Chronic Allograft Injury in Pediatric Renal Transplantation,” Journal of the American Society of Nephrology 21, no. 9 (2010): 1579–1586, 10.1681/ASN.2009111188. 20616168 PMC3013517

[19] A. Yamada , A. Tashiro , T. Hiraiwa , T. Komatsu , T. Kinukawa , and N. Ueda , “Long‐Term Outcome of Pediatric Renal Transplantation: A Single Center Study in Japan,” Pediatric Transplantation 18, no. 5 (2014): 453–462, 10.1111/petr.12299. 24931009

[20] B. Hocker , S. Zencke , K. Krupka , et al., “Cytomegalovirus Infection in Pediatric Renal Transplantation and the Impact of Chemoprophylaxis With (Val‐)ganciclovir,” Transplantation 100, no. 4 (2016): 862–870, 10.1097/TP.0000000000000888. 26736017

[21] R. Ettenger , H. Chin , K. Kesler , et al., “Relationship Among Viremia/Viral Infection, Alloimmunity, and Nutritional Parameters in the First Year After Pediatric Kidney Transplantation,” American Journal of Transplantation 17, no. 6 (2017): 1549–1562, 10.1111/ajt.14169. 27989013 PMC5445007

[22] Y. Gotoh , S. Shishido , Y. Hamasaki , et al., “Kidney Function of Japanese Children Undergoing Kidney Transplant With Preemptive Therapy for Cytomegalovirus Infection,” Transplant Infectious Disease 22, no. 3 (2020): e13271, 10.1111/tid.13271. 32108410

[23] L. Oomen , L. L. de Wall , E. A. M. Cornelissen , W. F. J. Feitz , and C. Bootsma‐Robroeks , “Prognostic Factors on Graft Function in Pediatric Kidney Recipients,” Transplantation Proceedings 53, no. 3 (2021): 889–896, 10.1016/j.transproceed.2020.10.017. 33257001

[24] T. Hussain , M. Burch , M. J. Fenton , et al., “Positive Pretransplantation Cytomegalovirus Serology Is a Risk Factor for Cardiac Allograft Vasculopathy in Children,” Circulation 115, no. 13 (2007): 1798–1805, 10.1161/CIRCULATIONAHA.106.627570. 17353448

[25] W. T. Mahle , M. T. Fourshee , D. M. Naftel , et al., “Does Cytomegalovirus Serology Impact Outcome After Pediatric Heart Transplantation?,” Journal of Heart and Lung Transplantation 28, no. 12 (2009): 1299–1305, 10.1016/j.healun.2009.07.011. 19783178

[26] G. Indolfi , N. Heaton , M. Smith , G. Mieli‐Vergani , and M. Zuckerman , “Effect of Early EBV and/or CMV Viremia on Graft Function and Acute Cellular Rejection in Pediatric Liver Transplantation,” Clinical Transplantation 26, no. 1 (2012): E55–E61, 10.1111/j.1399-0012.2011.01535.x. 21981027

[27] M. Furuichi , T. Fujiwara , A. Fukuda , M. Kasahara , and I. Miyairi , “Fulminant Hepatic Failure as a Risk Factor for Cytomegalovirus Infection in Children Receiving Preemptive Therapy After Living Donor Liver Transplantation,” Transplantation 100, no. 11 (2016): 2404–2409, 10.1097/TP.0000000000001435. 27495753

[28] L. A. Danziger‐Isakov , S. Worley , S. Arrigain , et al., “Increased Mortality After Pulmonary Fungal Infection Within the First Year After Pediatric Lung Transplantation,” Journal of Heart and Lung Transplantation 27, no. 6 (2008): 655–661, 10.1016/j.healun.2008.03.010. PMC2447528 18503966

[29] L. A. Danziger‐Isakov , S. Worley , M. G. Michaels , et al., “The Risk, Prevention, and Outcome of Cytomegalovirus After Pediatric Lung Transplantation,” Transplantation 87, no. 10 (2009): 1541–1548, 10.1097/TP.0b013e3181a492e8. 19461492 PMC2726779

[30] M. Liu , S. Worley , S. Arrigain , et al., “Respiratory Viral Infections Within One Year After Pediatric Lung Transplant,” Transplant Infectious Disease 11, no. 4 (2009): 304–312, 10.1111/j.1399-3062.2009.00397.x. 19422670 PMC7169860

[31] A. Perret , M. Le Marechal , R. Germi , et al., “Cytomegalovirus Detection Is Associated With ICU Admission in Non‐AIDS and AIDS Patients With Pneumocystis Jirovecii Pneumonia,” PLoS One 19, no. 1 (2024): e0296758, 10.1371/journal.pone.0296758. 38198473 PMC10781113

[32] R. D. Mosteller , “Simplified Calculation of Body‐Surface Area,” New England Journal of Medicine 317, no. 17 (1987): 1098, 10.1056/NEJM198710223171717. 3657876

[33] G. J. Schwartz , L. P. Brion , and A. Spitzer , “The Use of Plasma Creatinine Concentration for Estimating Glomerular Filtration Rate in Infants, Children, and Adolescents,” Pediatric Clinics of North America 34, no. 3 (1987): 571–590, 10.1016/s0031-3955(16)36251-4. 3588043

[34] M. D. Pescovitz , R. B. Ettenger , C. F. Strife , et al., “Pharmacokinetics of Oral Valganciclovir Solution and Intravenous Ganciclovir in Pediatric Renal and Liver Transplant Recipients,” Transplant Infectious Disease 12, no. 3 (2010): 195–203, 10.1111/j.1399-3062.2009.00478.x. 20002356

[35] W. Vaudry , R. Ettenger , P. Jara , et al., “Valganciclovir Dosing According to Body Surface Area and Renal Function in Pediatric Solid Organ Transplant Recipients,” American Journal of Transplantation 9, no. 3 (2009): 636–643, 10.1111/j.1600-6143.2008.02528.x. 19260840

[36] B. S. Clark , I. F. Chang , S. J. Karpen , et al., “Valganciclovir for the Prophylaxis of Cytomegalovirus Disease in Pediatric Liver Transplant Recipients,” Transplantation 77, no. 9 (2004): 1480, 10.1097/01.tp.0000123081.81022.33. 15167619

[37] A. Asberg , A. Bjerre , and M. Neely , “New Algorithm for Valganciclovir Dosing in Pediatric Solid Organ Transplant Recipients,” Pediatric Transplantation 18, no. 1 (2014): 103–111, 10.1111/petr.12179. 24152053 PMC3880615

[38] O. Peled , M. Berkovitch , E. Rom , et al., “Valganciclovir Dosing for Cytomegalovirus Prophylaxis in Pediatric Solid‐Organ Transplant Recipients: A Prospective Pharmacokinetic Study,” Pediatric Infectious Disease Journal 36, no. 8 (2017): 745–750, 10.1097/INF.0000000000001595. 28383392

[39] K. Jorga , B. Reigner , C. Chavanne , G. Alvaro , and N. Frey , “Pediatric Dosing of Ganciclovir and Valganciclovir: How Model‐Based Simulations Can Prevent Underexposure and Potential Treatment Failure,” CPT: Pharmacometrics & Systems Pharmacology 8, no. 3 (2019): 167–176, 10.1002/psp4.12363. 30354026 PMC6430157

[40] T. Nguyen , M. Oualha , C. Briand , et al., “Population Pharmacokinetics of Intravenous Ganciclovir and Oral Valganciclovir in a Pediatric Population to Optimize Dosing Regimens,” Antimicrobial Agents and Chemotherapy 65, no. 3 (2021): e02254‐20, 10.1128/AAC.02254-20. 33318012 PMC8092537

[41] A. N. Thomas , P. B. Nguyen , J. L. Miller , S. B. Neely , and T. V. Lewis , “Incidence of Cytomegalovirus DNAemia in Pediatric Post‐Renal Transplant Patients Receiving Weight‐Based vs Body Surface Area‐Based Valganciclovir Chemoprophylaxis,” Journal of Pediatric Pharmacology and Therapeutics 27, no. 2 (2022): 164–171, 10.5863/1551-6776-27.2.164. PMC8837208 35241989

[42] H. Wiltshire , C. V. Paya , M. D. Pescovitz , et al., “Pharmacodynamics of Oral Ganciclovir and Valganciclovir in Solid Organ Transplant Recipients,” Transplantation 79, no. 11 (2005): 1477–1483, 10.1097/01.tp.0000164512.99703.ad. 15940035

[43] G. J. Schwartz , A. Munoz , M. F. Schneider , et al., “New Equations to Estimate GFR in Children With CKD,” Journal of the American Society of Nephrology 20, no. 3 (2009): 629–637, 10.1681/ASN.2008030287. 19158356 PMC2653687

[44] G. J. Schwartz and D. F. Work , “Measurement and Estimation of GFR in Children and Adolescents,” Clinical Journal of the American Society of Nephrology 4, no. 11 (2009): 1832–1843, 10.2215/CJN.01640309. 19820136

[45] A. N. Mian and G. J. Schwartz , “Measurement and Estimation of Glomerular Filtration Rate in Children,” Advances in Chronic Kidney Disease 24, no. 6 (2017): 348–356, 10.1053/j.ackd.2017.09.011. 29229165 PMC6198668

[46] G. Varela‐Fascinetto , C. Benchimol , R. Reyes‐Acevedo , et al., “Tolerability of up to 200 Days of Prophylaxis With Valganciclovir Oral Solution and/or Film‐Coated Tablets in Pediatric Kidney Transplant Recipients at Risk of Cytomegalovirus Disease,” Pediatric Transplantation 21, no. 1 (2017): e12833, 10.1111/petr.12833. 27753183

[47] T. Lazzarotto , A. Chiereghin , A. Piralla , et al., “Kinetics of Cytomegalovirus and Epstein‐Barr Virus DNA in Whole Blood and Plasma of Kidney Transplant Recipients: Implications on Management Strategies,” PLoS One 15, no. 8 (2020): e0238062, 10.1371/journal.pone.0238062. 32841308 PMC7447038

[48] P. Ljungman , R. F. Chemaly , F. Khawaya , et al., “Consensus Definitions of Cytomegalovirus (CMV) Infection and Disease in Transplant Patients Including Resistant and Refractory CMV for Use in Clinical Trials: 2024 Update From the Transplant Associated Virus Infections Forum,” Clinical Infectious Diseases 79, no. 3 (2024): 787–794, 10.1093/cid/ciae321. 39041385 PMC11426271

[49] S. Chou , S. Alain , C. Cervera , et al., “Drug Resistance Assessed in a Phase 3 Clinical Trial of Maribavir Therapy for Refractory or Resistant Cytomegalovirus Infection in Transplant Recipients,” Journal of Infectious Diseases 229, no. 2 (2024): 413–421, 10.1093/infdis/jiad293. 37506264 PMC10873177

[50] R. K. Avery , S. Alain , B. D. Alexander , et al., “Maribavir for Refractory Cytomegalovirus Infections With or Without Resistance Post‐Transplant: Results From a Phase 3 Randomized Clinical Trial,” Clinical Infectious Diseases 75, no. 4 (2022): 690–701, 10.1093/cid/ciab988. 34864943 PMC9464078

[51] K. Sun , S. Hayes , C. Farrell , and I. H. Song , “Population Pharmacokinetic Modeling and Simulation of Maribavir to Support Dose Selection and Regulatory Approval in Adolescents With Posttransplant Refractory Cytomegalovirus,” CPT: Pharmacometrics & Systems Pharmacology 12, no. 5 (2023): 719–723, 10.1002/psp4.12943. 36789522 PMC10196418

